# Abdominal massage alleviates IBS-D by modulating the gut microbiota and suppressing the LPS/TLR4/NF-κB/MLCK pathway

**DOI:** 10.3389/fmicb.2026.1730607

**Published:** 2026-06-26

**Authors:** Huanan Li, Xiaoyu Wang, Lianjun Yin, Yusheng Li, Shun Fan, Haining Zhang, Wei Zhang, Haining Ou, Jingui Wang

**Affiliations:** 1 Department of Tuina, First Teaching Hospital of Tianjin University of Traditional Chinese Medicine, Tianjin, China; 2 National Clinical Research Center for Chinese Medicine, Tianjin, China; 3 Department of Rehabilitation, Guangdong Provincial Hospital of Traditional Chinese Medicine, Guangzhou, China; 4 The Postdoctoral Research Station, Guangdong Provincial Hospital of Chinese Medicine, Guangzhou, China; 5 Rehabilitation Medicine, The Third Affiliated Hospital of Southern Medical University, Guangzhou, China

**Keywords:** abdominal massage, fecal microbiota transplantation, gut microbiota, intestinal barrier function, irritable bowel syndrome-diarrhea, TLR4/MyD88/NF-κB pathway

## Abstract

**Background:**

Diarrhea-predominant irritable bowel syndrome (IBS-D) is a common functional gastrointestinal disorder with complex and incompletely understood pathophysiology. This study aimed to investigate the therapeutic effects and underlying mechanisms of abdominal massage on diarrhea-predominant IBS-D using a rat model.

**Methods:**

IBS-D was induced in Sprague–Dawley rats through a combination of maternal separation and chronic stress. The experimental interventions consisted of abdominal massage and fecal microbiota transplantation (FMT) using donor microbiota obtained from IBS-D + abdominal massage rats. Assessments included fecal moisture content (FMC), Bristol stool scores, visceral hypersensitivity, intestinal motility, open field test, gut microbiota, short-chain fatty acids (SCFAs), inflammatory markers (LPS, TLR4/MyD88/NF-κB pathway), and intestinal barrier integrity (TEM, tight junction proteins, FITC-dextran permeability).

**Results:**

Abdominal massage significantly improved diarrheal symptoms, visceral hypersensitivity, gastrointestinal motility, and anxiety-like behaviors in IBS-D rats. It restored gut microbiota diversity, reduced SCFA levels, and suppressed the TLR4/MyD88/NF-κB pathway, leading to decreased pro-inflammatory cytokines and LPS levels. FMT replicated these effects, suggesting the role of gut microbiota modulation. Moreover, abdominal massage also ameliorated barrier dysfunction in IBS-D rats by restoring ultrastructure, modulating MLCK and junctional proteins, and reducing macromolecular permeability.

**Conclusion:**

Abdominal massage alleviates IBS-D symptoms by modulating gut microbiota, inhibiting the TLR4/MyD88/NF-κB/MLCK signaling pathway, reducing inflammation, and restoring intestinal barrier function. These findings support its potential as a non-invasive therapeutic strategy for IBS-D.

## 1 Introduction

Diarrhea-predominant irritable bowel syndrome (IBS-D) is a highly prevalent functional gastrointestinal disorder characterized by chronic abdominal pain, altered bowel habits, and visceral hypersensitivity, significantly impairing patients’ quality of life (Altomare et al., 2021; AlDosari et al., 2024). Epidemiological studies indicate that IBS affects approximately 7–18% of the global adult population, with IBS-D accounting for about 28.8% of cases (Ford et al., 2010; Sperber et al., 2021). Despite its widespread clinical impact, the pathophysiology of IBS-D remains incompletely elucidated, and current therapeutic options are often limited in efficacy and associated with side effects (Choi et al., 2025; Ye et al., 2025). Growing evidence suggests that gut microbiota dysbiosis, low-grade mucosal inflammation, and impaired intestinal barrier function play pivotal roles in the development and persistence of IBS-D symptoms (Pittayanon et al., 2019; Hanning et al., 2021). In particular, the activation of the TLR4/MyD88/NF-κB signaling pathway has been implicated in promoting the release of pro-inflammatory cytokines and increasing intestinal permeability, thereby facilitating systemic immune activation and symptom manifestation (Hanning et al., 2021). Therefore, developing safe and effective therapeutic strategies should focus on modulating gut microbiota balance, inhibiting the TLR4/MyD88/NF-κB signaling pathway-mediated low-grade mucosal inflammation, and restoring intestinal barrier function, which holds significant scientific value and clinical relevance for alleviating abdominal pain, improving bowel habits, and reducing visceral hypersensitivity.

As an integral part of traditional medical systems, traditional Chinese medicine (TCM) offers a range of non-pharmacological therapies—such as acupuncture, massage, and moxibustion—that demonstrate considerable potential in alleviating clinical symptoms in patients with IBS-D (Wei et al., 2022). Abdominal massage applies specific manual techniques to the abdominal region to induce rhythmic passive movements, generating profound biomechanical oscillations and functional modulation of internal organs. This therapy not only regulates visceral qi and enhances intestinal motility but also mitigates gastrointestinal dysfunction associated with negative emotions such as anxiety and tension, thereby improving overall body resistance (Hanning et al., 2021; Wei et al., 2022; Zhang et al., 2025). As a distinctive branch of traditional Chinese massage, Jingu visceral massage is rooted in classical theory and refined through generations of clinical practice in the Tianjin region. Guided by the principle that “all diseases arise from qi disorder,” it focuses on abdominal manipulation to regulate the qi dynamics of the liver, spleen, and stomach, restoring physiological balance and relieving emotional stagnation through normalized qi flow (Yarandi and Christie, 2013; Komori et al., 2019). Precise control of manipulation strength and location facilitates water-dampness metabolism in the middle jiao and alleviates intestinal disorders related to liver qi invasion or spleen-stomach deficiency (Yarandi and Christie, 2013; Komori et al., 2019). Modern mechanistic studies indicate that abdominal massage can improve the gut microbiome, reduce abnormal intestinal mucosal permeability, and modulate autonomic nervous function, demonstrating multi-target potential for managing functional bowel disorders (Katiraei and Bultron, 2011). However, robust scientific evidence supporting the efficacy of abdominal massage and clarifying its molecular and microbiological mechanisms in IBS-D is still scarce. Furthermore, although fecal microbiota transplantation (FMT) has emerged as a promising strategy to correct microbial imbalances, its combined exploration with physical interventions like abdominal massage remains underexplored.

Therefore, this study aimed to systematically evaluate the therapeutic effects of abdominal massage in a well-established rat model of IBS-D induced by maternal separation and chronic stress, and to elucidate the underlying mechanisms. By demonstrating the efficacy and mechanistic basis of abdominal massage, this research may offer a scientific foundation for its clinical application as a safe, non-invasive complementary treatment for IBS-D, especially for patients with drug intolerance or a preference for physical therapy.

## 2 Materials and methods

### 2.1 Animals

A total of seven pregnant Sprague–Dawley (SD) rats (clean grade), averaging weighing 230 ± 10 g, were purchased from Beijing Vital River Laboratory Animal Technology Co., Ltd. (Beijing, China; Animal License Number: SCXK (Jing) 2021-0006). At postnatal day (PND) 39, all SD rats were randomly allocated into 11 experimental groups across three independent series: Series I (*n* = 10 per group): Control, IBS-D, IBS-D + abdominal massage groups; Series II (*n* = 8 per group): Control, IBS-D, IBS-D + abdominal massage. Series III (*n* = 8 per group): Control, IBS-D, IBS-D + abdominal massage, BS-D + saline solution, IBS-D + FMT groups.

The variation in sample size between Series I (*n* = 10) and Series II/III (*n* = 8) reflects a purpose-driven design balancing statistical requirements with ethical considerations. Sample size was determined by *a priori* power analysis using G*Power software, assuming a medium effect size (Cohen’s *f* = 0.25 ~ 0.5) based on preliminary experimental data (Huanan et al., 2024), with power = 0.80 and α = 0.05 for one-way ANOVA. The larger sample size in Series I was reserved for 16S rDNA sequencing analysis, accounting for the higher inter-individual variability inherent in microbiome data. Series II and III (*n* = 8) focused on behavioral, physiological, and molecular endpoints with lower measurement variability, where this sample size balanced statistical robustness with the 3R principle (Reduction). *Post-hoc* analysis confirmed that all primary outcomes achieved statistical significance, indicating adequate statistical power. Furthermore, one-way ANOVA is robust to minor group size imbalances when variance homogeneity is maintained, as verified by Levene’s test in our study.

All SD rats were housed at the specific pathogen-free (SPF) Grade Animal Experimental Center of Tianjin Hospital of Integrated Traditional Chinese and Western Medicine (Nankai Hospital), under controlled conditions: temperature maintained at 24 ± 2 °C, relative humidity between 40 and 75%, and a 12 h light/12 h dark cycle. Each rat was individually housed with ad libitum access to food and water, and bedding was changed daily to maintain a clean and hygienic environment. All animal experiments conducted in this study were reviewed and approved by the Animal Ethics Committee of Tianjin Hospital of Integrated Traditional Chinese and Western Medicine (Nankai Hospital) (Approval No.: NKYY-DWLL-2024-091). All procedures were performed in strict accordance with the Guidelines for the Ethical Treatment of Laboratory Animals.

### 2.2 Construction of the IBS-D rat model

**Figure 1:**
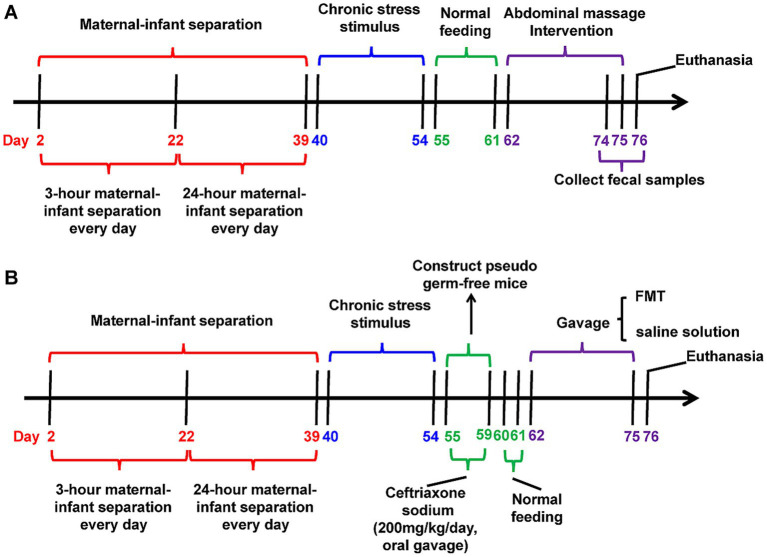
Schedule of the animal experiment. **(A)** Abdominal massage intervention in IBS-D model rats. **(B)** Fecal microbiota transplantation (FMT) in IBS-D model rats.

A total of 30 rats were randomly assigned using a random number table to one of three groups (Series I, *n* = 10 per group): the control group, the IBS-D group, and the IBS-D + abdominal massage group. In Series II, eight animals per group (Control, IBS-D, IBS-D + abdominal massage) were included. The animal model in Series II was established following the same protocol as described for Series I. The modeling procedure is illustrated in Figure 1A.

The IBS-D rat model was established using a combination of maternal separation and chronic stress stimulus. During the maternal separation procedure, neonatal SD rat pups were separated from their dams daily for 3 h (from 9:00 a.m. to 12:00 p.m.) from PND 2 to PND 21. During separation, they were placed in the same room but in separate plastic cages to prevent visual contact. From PND 22 onward, SD rat dams and pups were completely separated and individually housed in single cages; dams were maintained singly, while pups were assigned to cages based on their respective experimental groups. From the day of delivery of the SD rat dams, the animals had free access to water and food. The chronic stress stimuli were initiated once the SD rat pups had reached PND 39.

Chronic stress stimuli were initiated on PND 40 in the SD rat pups. The protocol consisted of three alternating stressors applied in a 3-day cycle over a total of 14 days (ending on PND 54): Tail Clip stress: A small butterfly clip (wrapped with cotton) was applied at 3 cm from the base of the tail for 3 min. Cold water immersion: Rats were placed in a transparent cylindrical water tank (15 ~ 18 cm deep) containing 5 °C ice water for 6 min. Restraint stress: Rats were restrained in a 5 cm diameter × 15 cm long plastic tube with tape securing the rear end to limit movement; they were restrained for 30 min daily. Following the 14-day stress regimen, all animals were maintained under standard housing conditions with free access to food and water for an additional 7 days. Control group rats were raised under conventional conditions without maternal separation or exposure to chronic stress.

### 2.3 Abdominal massage intervention in the IBS-D model rats

After being provided with regular food and water for a duration of 7 days, the rats in the IBS-D group underwent abdominal massage assays (IBS-D + abdominal massage group, PND 62) (Figure 1A). The core techniques of visceral massage, namely “abdominal pressing” and “abdominal rubbing,” were selected as the primary intervention methods. The specific operational procedures were as follows: The operator gently passed the left thumb through the lower left side of the rat’s forepaw and firmly grasped its upper body; the right hand was passed over the rat’s right hind limb, with the thenar eminence lightly pressing the hind limb, while the thumb was placed on the abdomen and the remaining four fingers were naturally positioned on the rat’s back to achieve effective immobilization.

The abdominal massage intervention, performed by a single trained operator to minimize variability, consisted of two core techniques: abdominal pressing and abdominal rubbing. All procedures followed a fixed protocol over 14 consecutive days (once daily, 20 min per session). For abdominal pressing: The Guanyuan (CV4) acupoint was rhythmically pressed with the thumb toward the pubic symphysis and spine. Pressure was calibrated by palpation of the abdominal aortic pulse to ensure consistent depth and safety. Upon detecting a slight arterial pulsation, pressure was initially maintained for 30 s, followed by an additional 1 min of sustained pressure. This cycle was repeated five times per session, resulting in a total duration of 10 min. For abdominal rubbing: Circular clockwise motions were performed around the Zhongwan (CV12) acupoint using the thenar eminence, at a frequency of 20–30 cycles/min, for 10 min.

Each of the aforementioned two techniques was administered once daily for a total of 10 min per session, over a continuous period of 14 days (PND 75). Rats in the healthy control group and IBS-D groups were raised under conventional feeding conditions without undergoing maternal separation or chronic stress procedures.

### 2.4 Fecal sample collection and 16S rRNA sequencing

In Series I, fecal samples were collected daily at 7:00 a.m. from rats in the Control, IBS-D, and IBS-D + abdominal massage groups (on PND 74, 75, 76). The standardized collection procedure was as follows: gloves, 75% ethanol, sterile cryovials, and markers were prepared in advance, and cryovials were arranged in sampling order. Within the SPF animal room, work surfaces were sprayed with 75% ethanol to maintain a relatively aseptic environment. Operators wore sterile gloves and performed all steps on UV-sterilized paper. Before each collection, hands and gloves were disinfected with 75% ethanol. Gentle lower-abdominal massage was applied to stimulate defecation, and 1–2 fresh fecal pellets were collected into sterile cryovials. Samples were immediately transferred to liquid nitrogen and subsequently stored at −80 °C. The entire process from collection to freezing was completed within 2 h. Samples were collected consecutively for 3 days per rat (on PND 74, 75, 76); the procedures for the second and third days followed the same protocol, and aliquots were stored frozen accordingly.

16S rRNA sequencing was performed using the Illumina MiSeq PE300 and NovaSeq PE250 platforms (OE Biotech, China). Detailed methods are available in Additional file 1.

### 2.5 Preparation of FMT donor material

FMT donor material was obtained from IBS-D model rats that received abdominal massage intervention in Series I (IBS-D + abdominal massage group, collected on PND 74, 75, 76). Donor sample collection was performed concurrently with the abdominal massage intervention. Briefly, fresh fecal samples were collected from the IBS-D + abdominal massage group (on PND 74, 75, 76) and immediately placed in sterile tubes. The samples were homogenized in sterile saline at a mass-to-volume ratio of 1:5 and filtered through a mesh screen to remove large particulate matter. The resulting filtrate was centrifuged at 3,000 rpm for 10 min, and the supernatant was discarded. The pellet was resuspended in saline and subjected to three repeated cycles of centrifugation under identical conditions. The final pellet was reconstituted in phosphate-buffered saline (PBS) to prepare a fecal bacterial suspension (10^8^ ~ 10^9^ CFU/mL) for FMT, which was stored at −80 °C until use.

### 2.6 Implementation of FMT in IBS-D model rats

The fecal bacterial suspension prepared from donor rats in the IBS-D + abdominal Massage group was administered to IBS-D recipient rats (i.e., the IBS-D + FMT group) via oral gavage. For this part of the experiment, a total of 40 male SD rats were stratified by body weight and randomly divided into five groups (*n* = 8 per group) using a lottery method (Series III): Control group, IBS-D group, IBS-D + abdominal massage group, IBS-D + FMT group, and IBS-D + saline solution group. Interventions for the Control, IBS-D model, and IBS-D + abdominal massage groups were performed as described in Sections 2.2 and 2.3 of this article.

The IBS-D + FMT group and IBS-D + saline solution group were treated according to the following protocol (Figure 1B): After successful induction of the IBS-D model, starting on PND 55, rats in these two groups received oral ceftriaxone pretreatment (200 mg/kg/day in a 1 mL volume) for 5 consecutive days (Rakoff-Nahoum et al., 2004; Dethlefsen and Relman, 2011) (Supplementary Figure S1). This regimen was designed to substantially perturb the gut microbiota without complete eradication, thereby minimizing the toxicity, systemic stress, and intestinal mucosal injury typically associated with broad-spectrum antibiotic combinations, while also avoiding undue interference with barrier function and inflammatory markers. The Control, IBS-D, and IBS-D + abdominal massage groups did not undergo this antibiotic pretreatment. Following a 2-day washout period (until PND 62), rats in the IBS-D + FMT group received daily oral gavage of the fecal bacterial suspension prepared as described in Section 2.5 for 14 consecutive days (until PND 75). Meanwhile, rats in the IBS-D + saline solution group were allowed free access to food and water and were administered a daily 2 mL saline oral gavage for 14 days (until PND 75). All procedures complied with the ARRIVE guidelines and standard FMT protocols (Borody et al., 2013) to ensure safety and reproducibility.

### 2.7 Fecal moisture content assessment

Fecal moisture content (FMC) was measured to objectively evaluate water content in rat feces. Fresh fecal samples were collected between 9:00 a.m. and 12:00 p.m. daily using metabolic cages where rats were individually housed for a 3-h period. After separating urine and contaminants using an acrylic tray, feces were weighed to obtain wet weight using an electronic balance. Samples were then dried in a preheated oven at 80 °C for 3 h and reweighed to determine dry weight. FMC was calculated as: (wet weight – dry weight) / wet weight × 100%. To minimize evaporation-related artifacts, samples were kept isolated from ambient air until weighing.

### 2.8 Bristol stool form scale

The Bristol stool form scale (Table 1) was used to assess the stool characteristics of rats within a 2-h period across different groups.

**Table 1:** Bristol stool trait scale.

Stool type	Stool form	Description
Type 1	Lumpy and hard	Separate, hard lumps that are difficult to pass; typically observed in severe constipation
Type 2	Lumpy and sausage-shaped	Sausage-shaped but lumpy and aggregated, with a lumpy surface; difficult to expel; indicative of constipation
Type 3	Sausage-shaped with cracks	Sausage-like in form with surface cracks; relatively easy to pass
Type 4	Sausage-shaped and smooth	Smooth, soft, sausage- or snake-shaped stool; typically observed in normal bowel function
Type 5	Soft blobs	Soft blobs with well-defined edges; can be passed easily
Type 6	Mushy stool	Fluffy, mushy pieces with ragged, irregular edges; unformed stool
Type 7	Watery stool	Entirely liquid, with no solid pieces; indicative of diarrhea

### 2.9 Abdominal withdrawal reflex score

Colorectal sensitivity was evaluated in different groups by measuring the abdominal withdrawal reflex (AWR) in response to graded colorectal distension (CRD) using a balloon catheter. Under isoflurane anesthesia, a lubricated balloon-tipped catheter was inserted into the colorectum approximately 7 cm from the anus and secured to the tail. After recovery and acclimation in a restraint chamber, the balloon was inflated incrementally to 20, 40, 60, and 80 mmHg, each maintained for 30 s and repeated three times with 3-min intervals. AWR scores (0–4) were recorded based on abdominal and postural responses, and the average score for each pressure was used for analysis. The average score from three trials was calculated for each pressure. AWR scoring criteria: 0 (No observable response to CRD stimulation); 1 (Mild head movement only in response to CRD); 2 (Visible contraction of the abdominal muscles); 3 (Abdominal contraction with lifting off the surface); 4 (Strong abdominal contraction leading to body arching).

### 2.10 Change rate of electromyographic integral

A Fr8 pediatric silicone catheter was connected to a three-way valve, a sphygmomanometer, and a pressure-controlled balloon. A 5-inch latex balloon was securely attached to the catheter and fastened. Under isoflurane anesthesia, rats were immobilized, and the lubricated catheter was inserted approximately 7 cm into the colorectum via the anus, then fixed at the tail base. Abdominal hair was shaved and disinfected, followed by a skin incision to expose the external oblique muscle. An electrode was implanted 1.5 cm above the inguinal ligament and connected to a PowerLab system. After the rats regained consciousness and stabilized, baseline electromyographic (EMG) activity was recorded over 30 s. The balloon was inflated sequentially to 20, 40, 60, and 80 mmHg, each maintained for 30 s and repeated three times with 3-min intervals. EMG signals were recorded throughout. The change in EMG integral was calculated as: [(integral during distension−baseline integral)/baseline integral] × 100%. After the procedure, the incision was sutured, and rats were singly housed for 7 days for recovery before subsequent experiments.

### 2.11 Assessment of colonic motility

Colonic motility was evaluated by measuring the expulsion time of an intrarectally implanted glass bead, along with fecal pellet output and weight. Before the experiment, rats were fasted for 24 h with free access to water. Under isoflurane anesthesia, a glass bead approximately 3 mm in diameter was inserted into the rectum approximately 3 cm from the anal verge. After full recovery from anesthesia, each rat was immediately placed in a metabolic cage, and the time required to expel the glass bead was recorded. Simultaneously, to assess fecal output, white absorbent paper was placed at the bottom of the metabolic cage to collect fecal pellets over a 72 h period. The number and weight of fecal pellets produced by each rat were measured. Throughout the assay, all animals had free access to both food and water.

### 2.12 Open field test

The open field test was conducted to assess exploratory behavior and anxiety-like states in rats. Animals were placed in a dark square arena (100 × 100 × 50 cm) divided into 5 × 5 grids, with a defined central zone. After 30 min of acclimation under controlled conditions, each rat was placed in the center and allowed to explore freely for 5 min. Behaviors recorded included grid crossings, rears, grooming episodes, and central zone entries/duration. The arena was cleaned with 75% ethanol between trials to remove odor cues. Key outcomes comprised total crossings, rears, grooming frequency, and central zone activity ratio, reflecting exploration and anxiety levels.

### 2.13 Analysis of short-chain fatty acids

Fecal samples from the different groups (PND 76) were accurately weighed under low-temperature conditions, homogenized, and mixed with extraction solvent to isolate short-chain fatty acids (SCFAs). A standard curve was established using SCFA calibration solutions at various concentrations. Both standards and extracted samples were analyzed under identical conditions by gas chromatography–mass spectrometry (GC–MS). SCFA concentrations in the samples were quantified based on the standard curve and normalized to the actual content per mass of fecal sample. Detailed methods are available in Additional file 1.

### 2.14 Lipopolysaccharide analysis

Fecal samples were collected from the cecum of rats, weighed (≥50 mg), and homogenized in 10% (w/v) PBS (0.05 mol/L, pH 7.2–7.4) using a tissue homogenizer on ice. The homogenate was centrifuged at 5,000 rpm for 15 min, and the supernatant was collected for lipopolysaccharide (LPS) analysis. Standards and samples were loaded in duplicate onto the enzyme immunoassay plate, with the latter being diluted 5-fold before assay. After adding the enzyme conjugate to all except blank wells, the plate was incubated at 37 °C for 60 min, washed five times, and then incubated with chromogenic substrates A and B for 15 min at 37 °C in the dark. The reaction was stopped with sulfuric acid, and the optical density was measured at 450 nm within 15 min. LPS concentrations were determined based on a standard curve derived from known standards.

### 2.15 Western-blot assay

Ileal tissue samples from rats were weighed and homogenized in RIRP lysis buffer (BN25001-A, Bairuiji) at a 1:6 (w/v) ratio on ice, followed by intermittent vortexing and centrifugation at 4 °C. The supernatant was aliquoted and stored at −80 °C. Protein concentration was determined using a BCA method (BCA Protein Assay Kit, SL201, UtiBody) with a standard curve for quantification. After denaturation, protein samples were separated by SDS-PAGE with a 10% separating gel and a 5% stacking gel, and subsequently transferred onto a PVDF membrane. The membrane was blocked with 5% skim milk for 2 h, followed by incubation with primary antibody at 4 °C overnight and corresponding secondary antibody at room temperature for 1.5 h. Finally, protein bands were detected using an ECL reagent and visualized under a chemiluminescence imaging system (ChemiScope6100, Qinxiang). Band densities were quantified with ImageJ software using β-actin as the internal reference. The following primary antibodies were used: TLR4 (48-2300, Thermo Fisher), MyD88 (PA5-19919, Thermo Fisher), NF-κB p65 (PA5-16545, Thermo Fisher), MYLK (21642-1-AP, Proteintech), F-Actin (PC1571S, Abmart), Occludin (27260-1-AP, Proteintech), E-cadherin (20874-1-AP, Proteintech), ZO-1 (21773-1-AP, Proteintech), and phospho-Myosin Light Chain (Ser20) (PU198546S, Abmart). The secondary antibody was Goat Anti-Rabbit IgG (H&L) conjugated to HRP (ab205718, Abcam).

### 2.16 Enzyme-linked immunosorbent assay

Ileal tissue samples from rats were homogenized in PBS at a weight-to-volume ratio of 1:9, followed by centrifugation to collect the supernatant. A series of five standard solutions was prepared using sequential twofold dilutions. The microplate was configured with blank wells, standard wells, and sample wells. Each sample well received 40 μL of diluent and 10 μL of the test sample, yielding a total volume of 50 μL with a fivefold dilution. After incubation at 37 °C for 30 min, the plate was washed five times. Enzyme conjugate was added to all wells except the blanks, followed by another incubation and washing step. Subsequently, 50 μL of chromogen A and 50 μL of chromogen B were added to each well, mixed gently, and incubated at 37 °C in the dark for 10 min. The reaction was terminated by adding 50 μL of stop solution. Optical density was measured at 450 nm using the blank wells for zeroing.

### 2.17 Fluorescein isothiocyanate-dextran tracer technique

Intestinal barrier permeability was assessed using the fluorescein isothiocyanate (FITC)-dextran tracer technique. A standard curve was generated from serially diluted FITC-dextran standards. Isolated rat ileal segments were ligated at both ends and injected with FITC-dextran solution (HY-128868, MCE). Fluorescence intensity was measured with a fluorescence spectrophotometer (F95S, Shanghai Precision Instrument Co., Ltd) at 480 nm excitation and 520 nm emission. The concentration of FITC-dextran in portal blood was determined using a standard curve.

### 2.18 Ultrastructural analysis of rat intestinal mucosa by transmission electron microscopy

The ultrastructure of rat intestinal mucosa was observed using transmission electron microscopy (TEM). Briefly, tissue samples (≈1 mm^3^) were fixed in 2.5% glutaraldehyde (Spi-Chem, United States) and post-fixed with 1% osmium tetroxide (Ted Pella Inc., United States). After graded ethanol dehydration, the specimens were infiltrated with epoxy resin, embedded, and polymerized. Semi-thin sections were first prepared for orientation, followed by ultrathin sectioning (70 nm). The sections were then double-stained with uranyl acetate and lead citrate, and examined under a TEM (JEM1400, JEOL, Japan) for imaging.

### 2.19 Statistical analysis

This study used GraphPad Prism 10.0 for statistical analysis of experimental data. For quantitative data, normality and homogeneity of variance were first tested. If *p* > 0.05, data were expressed as mean ± standard deviation (mean ± SD), and inter-group differences were evaluated using one-way ANOVA followed by LSD-t test. To address multiple comparisons and control the Type I error rate, *p*-values from LSD-t tests were adjusted using Bonferroni correction. A djusted *p*-values (p.adj) < 0.05 were considered statistically significant. If data did not meet normality or homogeneity assumptions, they were described using median (lower quartile, upper quartile) and analyzed with non-parametric tests. For non-parametric multi-group comparisons, Kruskal-Wallis test was applied, followed by Dunn’s post-hoc test with Bonferroni adjustment. The statistical significance level was set at *p* < 0.05.

## 3 Results

### 3.1 Abdominal massage ameliorates diarrhea, visceral hypersensitivity, gastrointestinal motility, and anxiety-like behaviors in IBS-D rats

**Figure 2:**
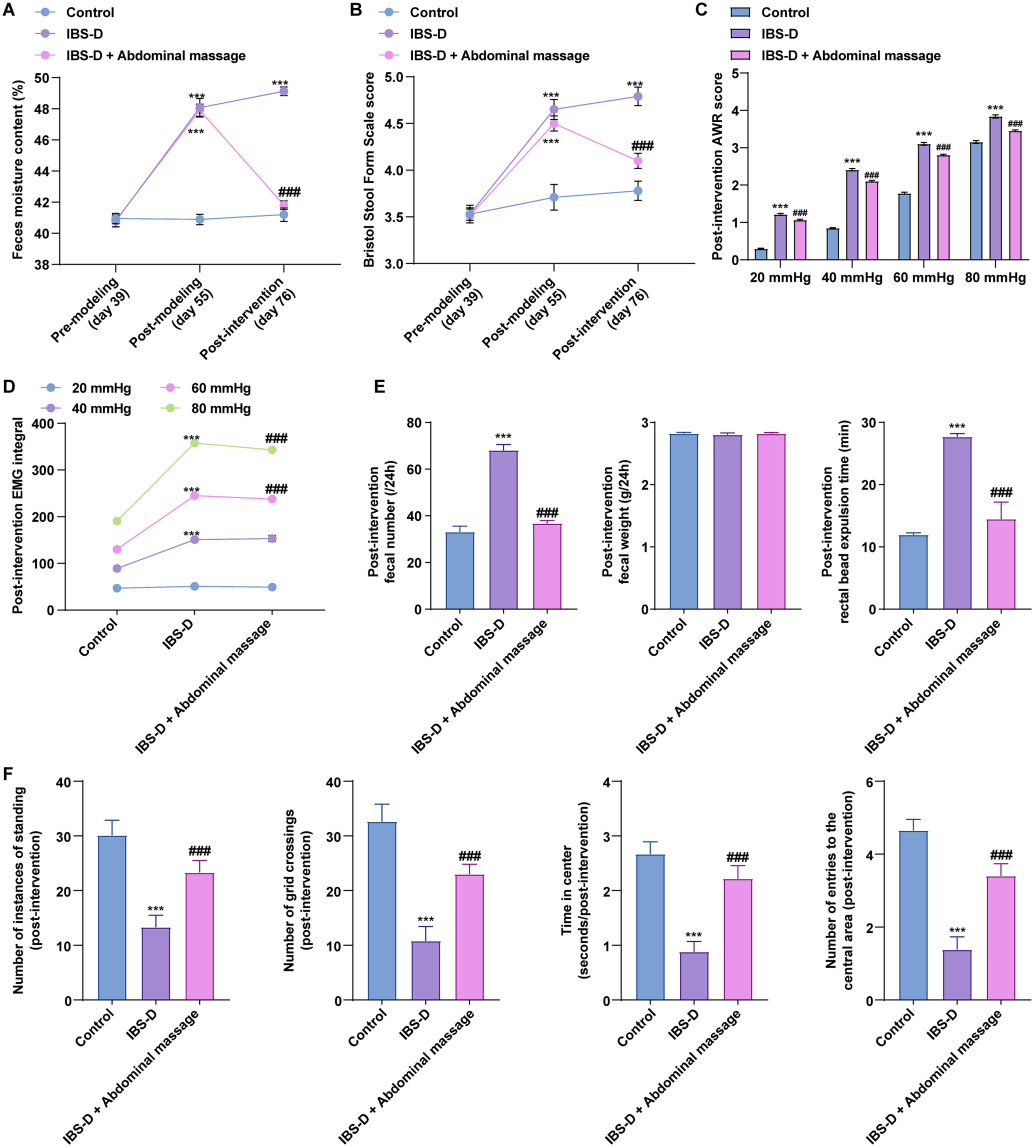
Abdominal massage ameliorates diarrhea, visceral hypersensitivity, gastrointestinal motility, and anxiety-like behaviors in IBS-D rats. **(A,B)** The fecal moisture content (FMC) and Bristol scores among the control, IBS-D, and IBS-D + abdominal massage groups at pre-modeling (PND 39), post-modeling (PND 55), and post-intervention (PND 76). **(C,D)** The abdominal withdrawal reflex (AWR) scores and electromyographic (EMG) integral among the control, IBS-D, and IBS-D + abdominal massage groups under different colorectal distention (CRD) pressure levels at post-intervention. **(E)** The fecal number, fecal weight, and rectal bead expulsion time among the control, IBS-D, and IBS-D + abdominal massage groups at post-intervention. **(F)** The number of instances of standing, grid crossings, entries to the central area, and the time in the center among the control, IBS-D, and IBS-D + abdominal massage groups at post-intervention. ^***^p < 0.001 for IBS-D vs. control; ^###^*p* < 0.001 for IBS-D + abdominal massage vs. IBS-D.

We first analyzed the FMC and Bristol scores in rats across the control, IBS-D, and IBS-D + abdominal massage groups at three stages: pre-modeling (PND 39), post-modeling (PND 55), and post-intervention (PND 76). As shown in Figures 2A,B, at pre-modeling, no significant differences were observed in FMC and Bristol scores among the control, IBS-D, and IBS-D + abdominal massage groups. At post-modeling, both the IBS-D and IBS-D + abdominal massage groups exhibited significantly increased FMC and Bristol scores compared to the control group (*p* < 0.001; Figures 2A,B). At the post-intervention, the IBS-D + abdominal massage group showed a significant reduction in FMC and Bristol scores compared to the IBS-D group (*p* < 0.001, Figures 2A,B). These results indicate that rats in the IBS-D group displayed marked diarrheal symptoms, and abdominal massage intervention significantly ameliorated diarrhea in IBS-D rats.

We next compared the AWR scores and EMG integral change rates among the control, IBS-D, and IBS-D + abdominal massage groups at post-modeling and post-intervention. At post-modeling, under CRD of 40, 60, and 80 mmHg, both the IBS-D and IBS-D + abdominal massage groups showed significantly higher AWR scores and EMG integral change rates compared to the control group (Supplementary Figures S2A,B). However, no significant differences were observed between the IBS-D and IBS-D + abdominal massage groups at this stage (Supplementary Figures S2A,B). At post-intervention, the IBS-D + abdominal massage group exhibited a significant reduction in AWR scores across all CRD pressures compared to the IBS-D model group (Figure 2C). Compared to the control group, the IBS-D + abdominal massage group exhibited significantly lower EMG integral change rates at 60 and 80 mmHg CRD pressures (Figure 2D). In addition, At post-modeling, compared to the control group, both the IBS-D and IBS-D + abdominal massage groups exhibited prolonged fecal number and rectal bead expulsion time, although no significant difference was observed in stool weight (Supplementary Figure S2C). At post-intervention, compared to the IBS-D group, the IBS-D + abdominal massage group exhibited a reduction in fecal number and rectal bead expulsion time, although no significant change was observed in fecal weight (Figure 2E). These findings suggest that abdominal massage primarily modulates the motility dimension by regulating intestinal transit rhythm, shifting defecation patterns from rapid and frequent to more regular and moderate, without altering the overall volume of intestinal contents.

The open field test showed that, at post-modeling, both the IBS-D and IBS-D + abdominal massage groups exhibited significant reductions in the number of instances of standing, grid crossings, entries to the central area, and the time in the center compared to the control group (Supplementary Figure S2D). At post-intervention, the IBS-D + abdominal massage group demonstrated significant improvements in all these behavioral dimensions relative to the IBS-D group (Figure 2F). These results suggest that abdominal massage significantly ameliorates visceral hypersensitivity, normalizes intestinal motility, and alleviates anxiety- and depression-like behaviors in IBS-D rats.

### 3.2 Abdominal massage modulates the gut microbiota structure in IBS-D rats

**Figure 3:**
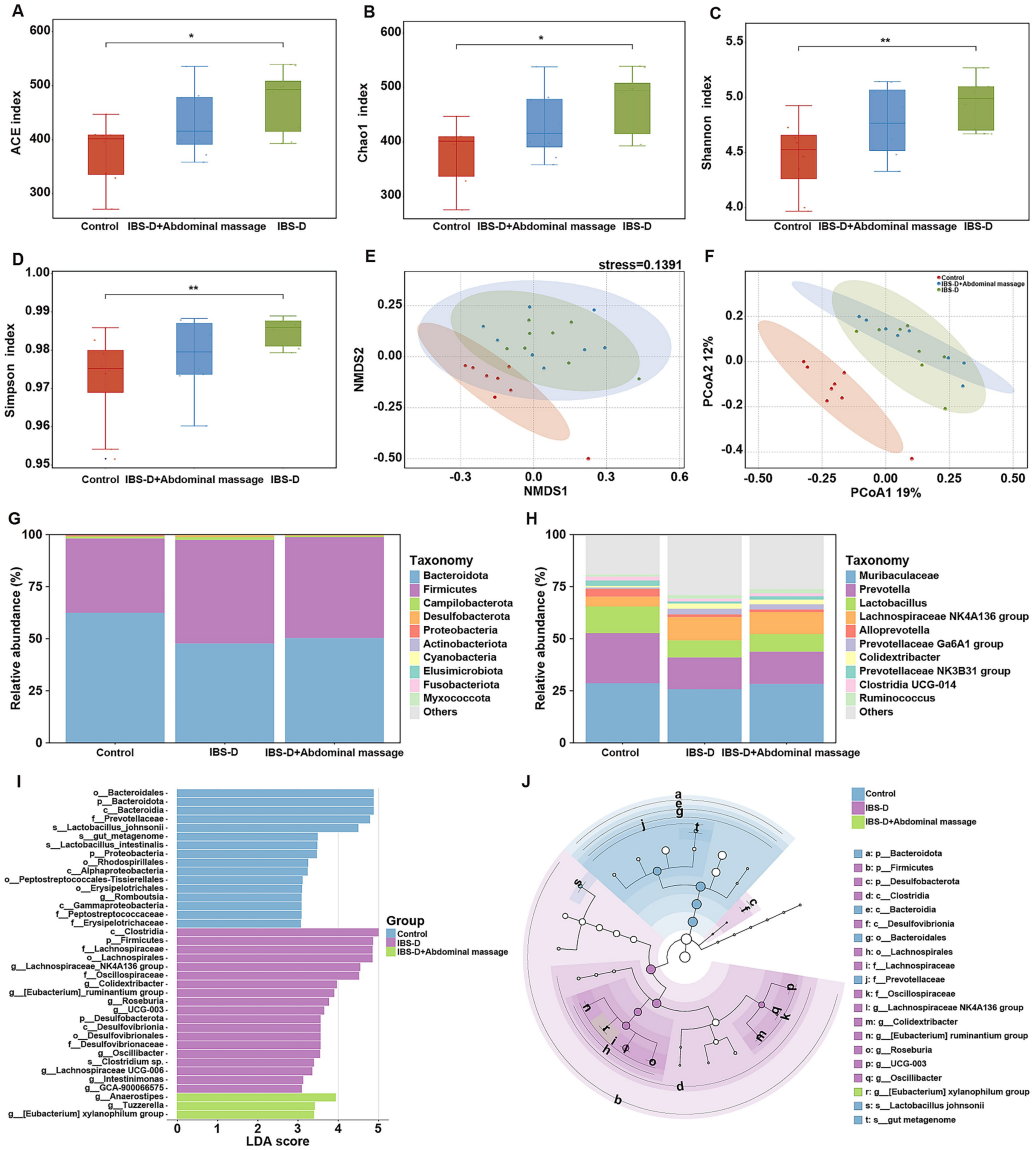
Abdominal massage modulates the gut microbiota structure in IBS-D rats. **(A–D)** The ACE, Chao1, Shannon, and Simpson indices in control, IBS-D, and IBS-D + abdominal massage groups. **(E)** NMDS analysis based on Bray-Curtis distances. **(F)** Principal coordinate analysis. **(G,H)** The microbial composition among the control, IBS-D, and IBS-D + abdominal massage groups at the phylum and genus levels. **(I,J)** Biomarker identification among different treatment groups was performed based on LefSe analysis with an LDA score threshold of >3. *p < 0.05, **p < 0.01.

To investigate the impact of abdominal massage on the gut microbiota in IBS-D rats, fecal samples were collected from the IBS-D + abdominal massage group and subjected to 16S rRNA sequencing analysis. Alpha diversity showed that compared to the control group, the ACE index, Chao1 index, Shannon index, and Simpson index were significantly increased in the IBS-D group (Figures 3A–D). However, these indices were reduced in the IBS-D + abdominal massage group relative to the IBS-D group (Figures 3A–D). NMDS analysis revealed a partial separation between the control group and the IBS-D or IBS-D + abdominal massage groups (Figure 3E). In contrast, PCoA analysis clearly demonstrated distinct clustering of samples from the control, IBS-D, and IBS-D + abdominal massage groups (Figure 3F). These results indicate that, compared to the control group, rats with IBS-D exhibited significant alterations in gut microbial composition, while abdominal massage partially restored the gut microbiota structure.

We analyzed the differences in microbial composition among the control, IBS-D, and IBS-D + abdominal massage groups at both the phylum and genus levels. At the phylum level, Bacteroidota and Firmicutes were the most abundant phyla across all three groups. Compared to the control group, the IBS-D group exhibited a decreased relative abundance of Bacteroidota and an increase in Firmicutes after model establishment. Abdominal massage intervention increased the abundance of Bacteroidota in IBS-D rats (Figure 3G). At the genus level, the top three genera were *Muribaculaceae*, *Prevotella*, and *Lactobacillus* (Figure 3H). The control group showed a higher relative abundance of *Lactobacillus*. In contrast, the abundances of *Prevotella* and *Lactobacillus* were decreased in both the IBS-D and IBS-D + abdominal massage groups compared to the control, while the abundance of *Lachnospiraceae_NK4A136*_group was increased in these two groups (Figure 3H).

Linear Discriminant Analysis Effect Size (LEfSe) analysis identified *Romboutsia* and *Lactobacillus* as microbial biomarkers in the control group. In the IBS-D group, biomarkers included *Lachnospiraceae_NK4A136_group*, *[Eubacterium]_ruminantium_group*, *Roseburia*, *Lachnospiraceae_UCG-006*, *Intestinimonas*, *GCA-900066575*, *UCG-003*, and *Oscillibacter*. The IBS-D + abdominal massage group was characterized by biomarkers such as *Anaerostipes*, *Tuzzerella*, and *[Eubacterium]_xylanophilum_group* (Figure 3I). Further taxonomic analysis revealed that biomarkers in the control group were predominantly affiliated with the phylum Bacteroidota, whereas those in the IBS-D and IBS-D + abdominal massage groups were mainly associated with Desulfobacterota (Figure 3J). These findings indicate distinct phylogenetic clustering of microbial biomarkers among the three groups.

### 3.3 Abdominal massage may suppress the production of SCFAs in the intestines of IBS-D rats

**Figure 4:**
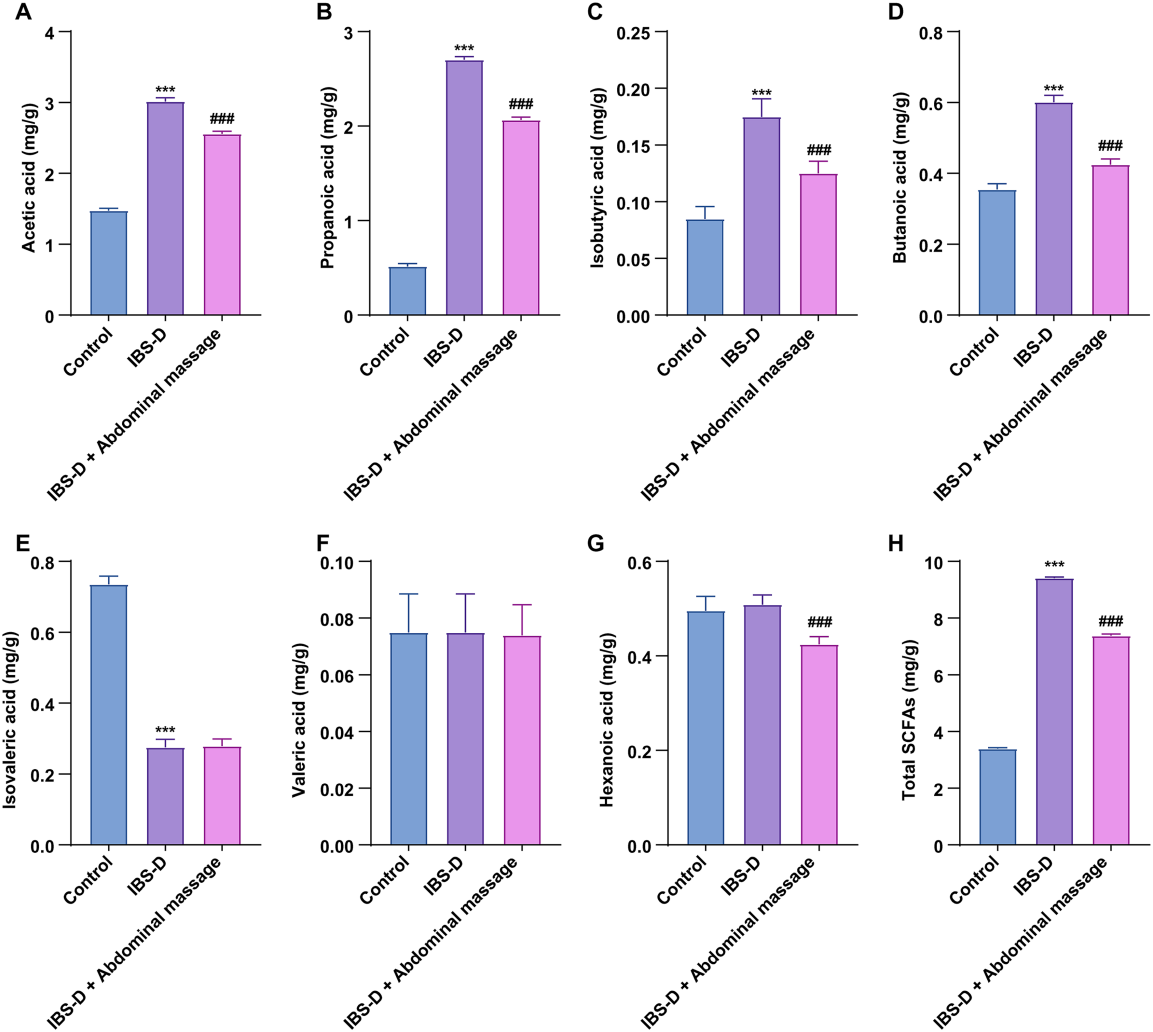
Abdominal massage may suppress the production of SCFAs in the intestines of IBS-D rats. **(A–G)** The levels of acetic acid, propanoic acid, isobutyric acid, butanoic acid, isovaleric acid, valeric acid, and hexanoic acid in control, IBS-D, and IBS-D + abdominal massage groups. **(H)** The levels of total short-chain fatty acids in control, IBS-D, and IBS-D + abdominal massage groups. ^***^p < 0.001 for IBS-D vs. control; ^###^*p* < 0.001 for IBS-D + abdominal massage vs. IBS-D.

Studies have demonstrated that Lactobacillus plays a crucial role in fermenting indigestible complex carbohydrates, leading to the production of substantial metabolites such as SCFAs (Anumudu et al., 2024; Terpou et al., 2025). In this study, the higher abundance of *Lactobacillus* observed in the control group suggests its potential association with the maintenance of intestinal health (Figure 3H), identifying it as a core beneficial member of the gut microbiota. Therefore, we investigated the effects of abdominal massage on intestinal SCFA levels in IBS-D rats. As shown in Figures 4A–H, compared to the control group, IBS-D rats exhibited significantly increased levels of acetic acid, propanoic acid, isobutyric acid, butanoic acid, and total SCFAs in fecal samples, whereas no significant differences were observed in the levels of valeric acid and hexanoic acid. The IBS-D + abdominal massage group showed markedly reduced concentrations of acetic acid, propanoic acid, isobutyric acid, butanoic acid, and total SCFAs compared to the IBS-D group, while the levels of isovalerate and valerate remained statistically unchanged (Figures 4A–H). These results indicate that abdominal massage may suppress the production of SCFAs in the intestines of IBS-D rats.

### 3.4 FMT ameliorates visceral hypersensitivity, gut dysmotility, and anxiety/depression-like behaviors via suppressing SCFA in IBS-D rats

**Figure 5:**
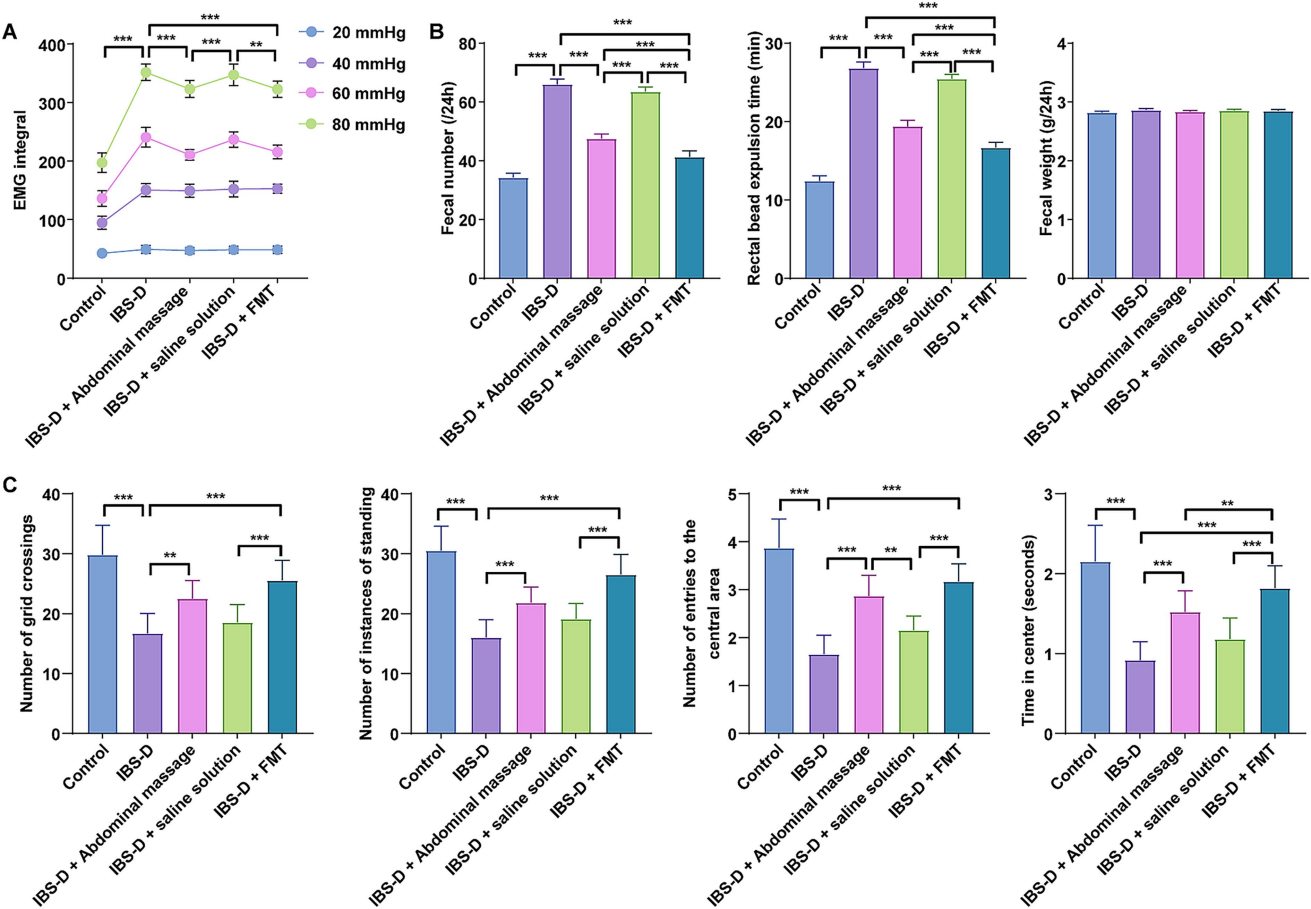
FMT ameliorates visceral hypersensitivity, gut dysmotility, and anxiety/depression-like behaviors in IBS-D rats. **(A)** The electromyographic (EMG) integral among the control, IBS-D, and IBS-D + abdominal massage, IBS-D + saline solution, and IBS-D + FMT groups. **(B)** The fecal number, rectal bead expulsion, and fecal weight time among the control, IBS-D, and IBS-D + abdominal massage, IBS-D + saline solution, and IBS-D + FMT groups. **(C)** The number of grid crossings, instances of standing, entries to the central area, and the time in the center among the control, IBS-D, and IBS-D + abdominal massage, IBS-D + saline solution, and IBS-D + FMT groups. ^*^p < 0.01, ^***^p < 0.001.

To further investigate whether the abdominal massage-induced gut microbiota alterations contribute to the therapeutic effects in IBS-D rats, we performed FMT from donors in the IBS-D + abdominal massage group to recipient IBS-D rats. Under 80 mmHg CRD stimulation, the EMG integral change rate was significantly decreased in the IBS-D + abdominal massage (vs. the IBS-D group) and IBS-D + FMT (vs. IBS-D + saline solution) groups, again with no significant differences between the two treatment groups (Figure 5A). Both the IBS-D + abdominal massage (vs. IBS-D group) and IBS-D + FMT (vs. IBS-D + saline solution) groups exhibited shortened fecal number and rectal bead expulsion time (Figure 5B), and the IBS-D + FMT group showed lower fecal number and rectal bead expulsion time compared to the IBS-D + abdominal massage group (Figure 5B). In the open field test, the number of grid crossings, instances of standing, entries to the central area, and time in the center were all significantly increased in the IBS-D + abdominal massage (vs. IBS-D group) and IBS-D + FMT (vs. IBS-D + saline solution) groups (Figure 5C). Notably, the time in the center was further significantly elevated in the IBS-D + FMT group compared to the IBS-D + abdominal massage group (Figure 5C). These results demonstrate that FMT ameliorates visceral hypersensitivity, normalizes intestinal motility, and attenuates anxiety- and depression-like behaviors in IBS-D rats.

**Figure 6:**
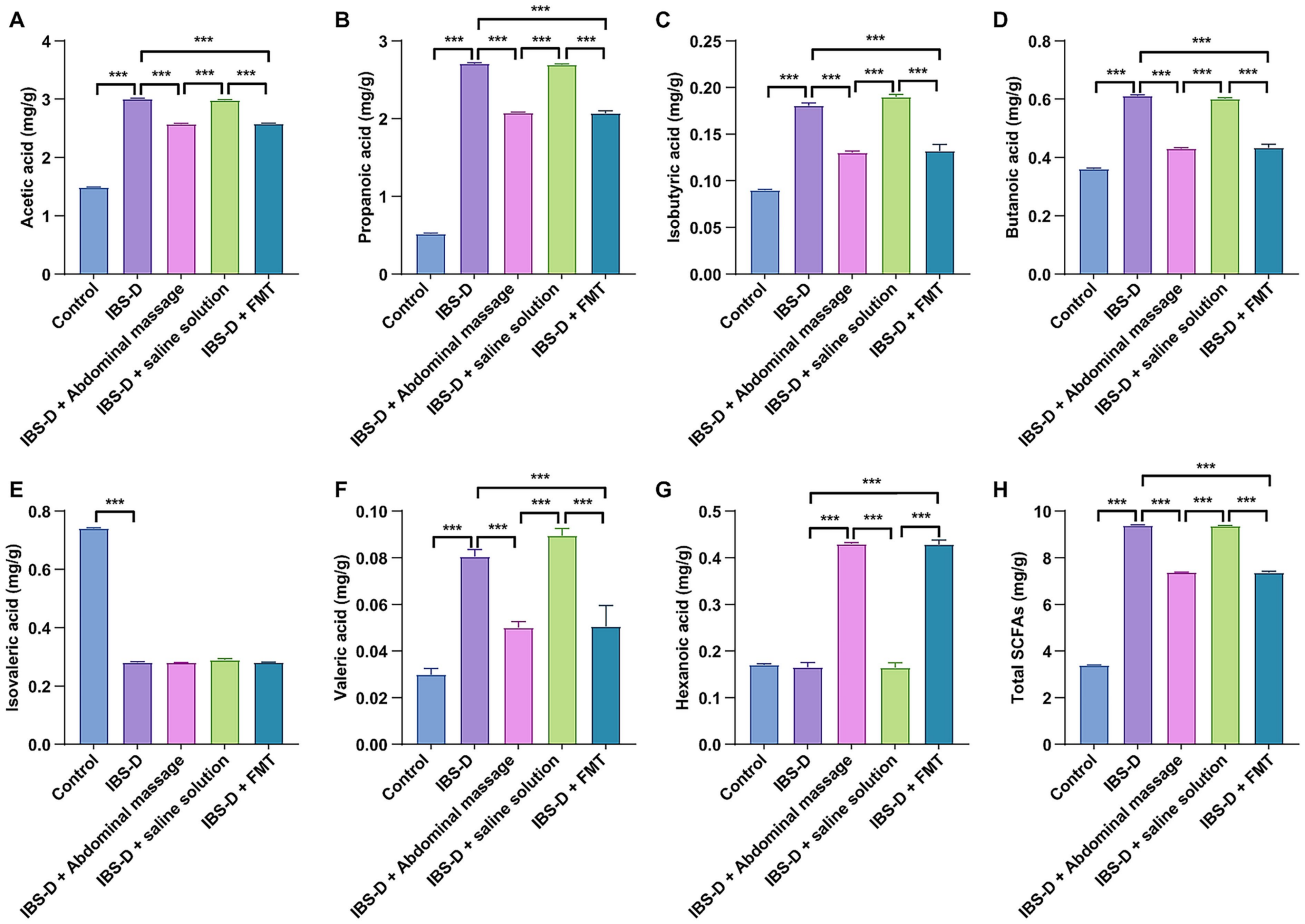
FMT may suppress the production of SCFAs in the intestines of IBS-D rats. **(A–G)** The levels of acetic acid, propanoic acid, isobutyric acid, butanoic acid, isovaleric acid, valeric acid, and hexanoic acid in control, IBS-D, and IBS-D + abdominal massage, IBS-D + saline solution, and IBS-D + FMT groups. **(H)** The levels of total short-chain fatty acids in control, IBS-D, and IBS-D + abdominal massage, IBS-D + saline solution, and IBS-D + FMT groups. ***p < 0.001.

Moreover, both the abdominal massage (vs. IBS-D) and FMT (vs. IBS-D + saline solution) intervention groups showed significantly decreased fecal concentrations of acetic acid, propanoic acid, isobutyric acid, butanoic acid, valeric acid, and SCFAs (Figures 6A–H). In contrast, hexanoic acid levels were significantly elevated in both treatment groups. No statistically significant differences were observed in any of these SCFA parameters between the abdominal massage and FMT groups (Figures 6A–H). These findings suggest that FMT significantly suppresses SCFA production in IBS-D rats.

### 3.5 Inhibition of the TLR4/MyD88/NF-κB pathway may mediate the therapeutic effects of abdominal massage and FMT in IBS-D rats

**Figure 7:**
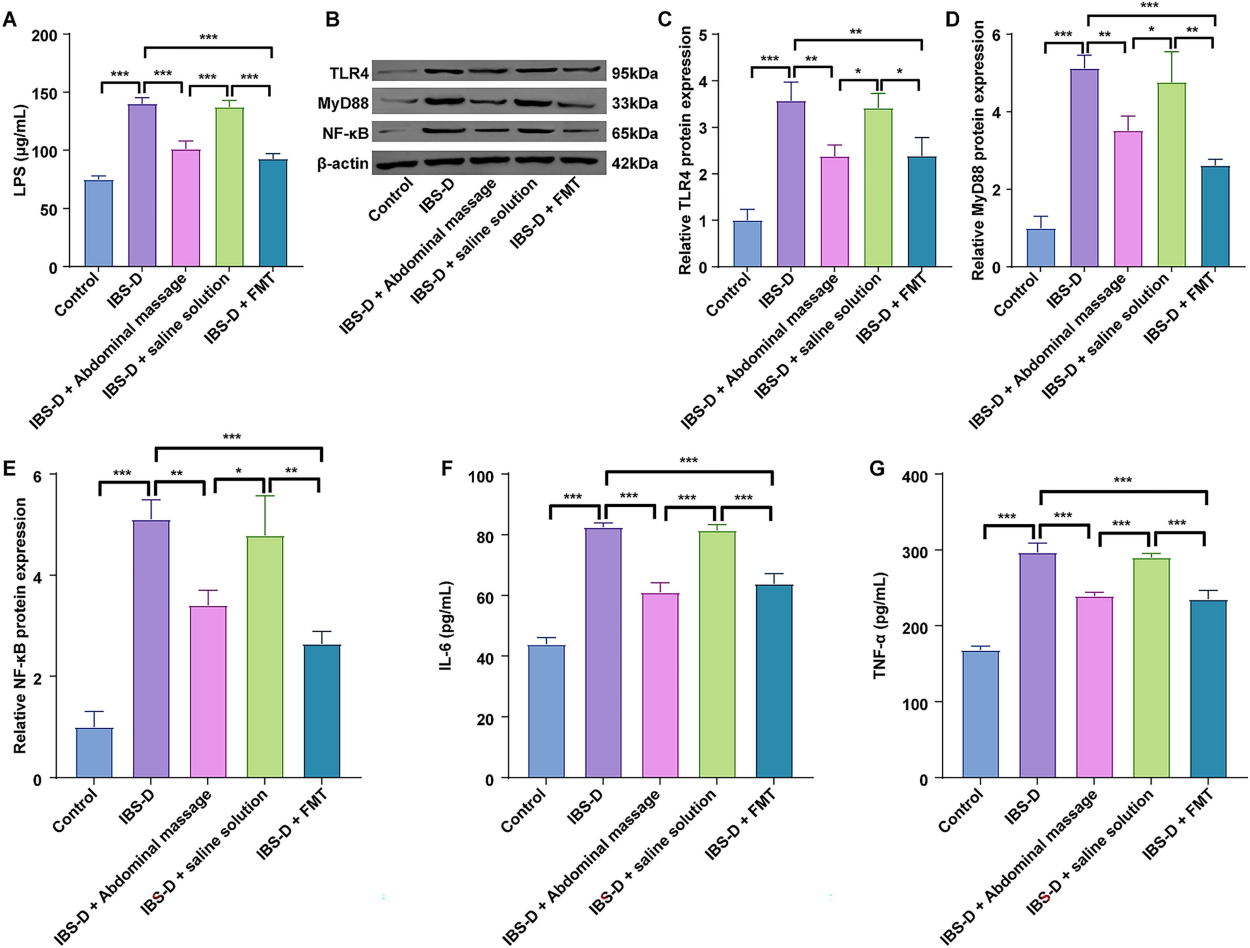
Inhibition of the TLR4/MyD88/NF-κB pathway may mediate the therapeutic effects of abdominal massage and FMT in IBS-D rats. **(A)** The lipopolysaccharide (LPS) levels in the control, IBS-D, and IBS-D + abdominal massage, IBS-D + saline solution, and IBS-D + FMT groups. **(B–E)** The levels of TLR4, MyD88, and NF-κB protein expression in the control, IBS-D, and IBS-D + abdominal massage, IBS-D + saline solution, and IBS-D + FMT groups. **(F,G)** The levels of IL-6 and TNF-α in the control, IBS-D, and IBS-D + abdominal massage, IBS-D + saline solution, and IBS-D + FMT groups. *p < 0.05, **p < 0.01, ***p < 0.001.

Dysbiosis of the gut microbiota can sequentially activate the TLR4/MyD88/NF-κB signaling pathway, leading to the release of pro-inflammatory cytokines such as IL-1, IL-6, and TNF-α. This process induces intestinal inflammation, increases gut permeability, and facilitates the translocation of bacteria, endotoxins, and macromolecules into the systemic circulation (Rong et al., 2021). Consequently, elevated levels of LPS contribute to systemic chronic low-grade inflammation (Zhang, 2025). Therefore, we next evaluated the effect of abdominal massage and FMT on the levels of LPS, the TLR4/MyD88/NF-κB signaling pathway, and pro-inflammatory cytokines in IBS-D rats. Compared to the control group, the ileal LPS levels were markedly elevated in IBS-D rats, an alteration that was significantly reversed by abdominal massage (vs. IBS-D group) and FMT (vs. IBS-D + saline solution) (Figure 7A). As shown in Figures 7B–E, the protein expression levels of TLR4, MyD88, and NF-κB were significantly upregulated in the ileal tissues of IBS-D model rats compared with the control group. Both abdominal massage (vs. IBS-D group) and FMT (vs. IBS-D + saline solution) significantly suppressed the expression of these proteins in IBS-D rats (Figures 3, 7B–E). Moreover, compared to the control group, the levels of pro-inflammatory cytokines (IL-6 and TNF-α) were significantly elevated in the ileal tissue of IBS-D rats (Figures 7F,G). Both abdominal massage (vs. IBS-D) and FMT (vs. IBS-D + saline solution) interventions markedly suppressed the expression of these pro-inflammatory cytokines (Figures 7F,G). These findings suggest that abdominal massage and FMT may alleviate symptoms in IBS-D rats by modulating the gut microbiota and reducing intestinal inflammation, potentially through inhibiting the activation of the TLR4/MyD88/NF-κB signaling pathway.

### 3.6 Abdominal massage ameliorates intestinal barrier dysfunction in IBS-D rats

**Figure 8:**
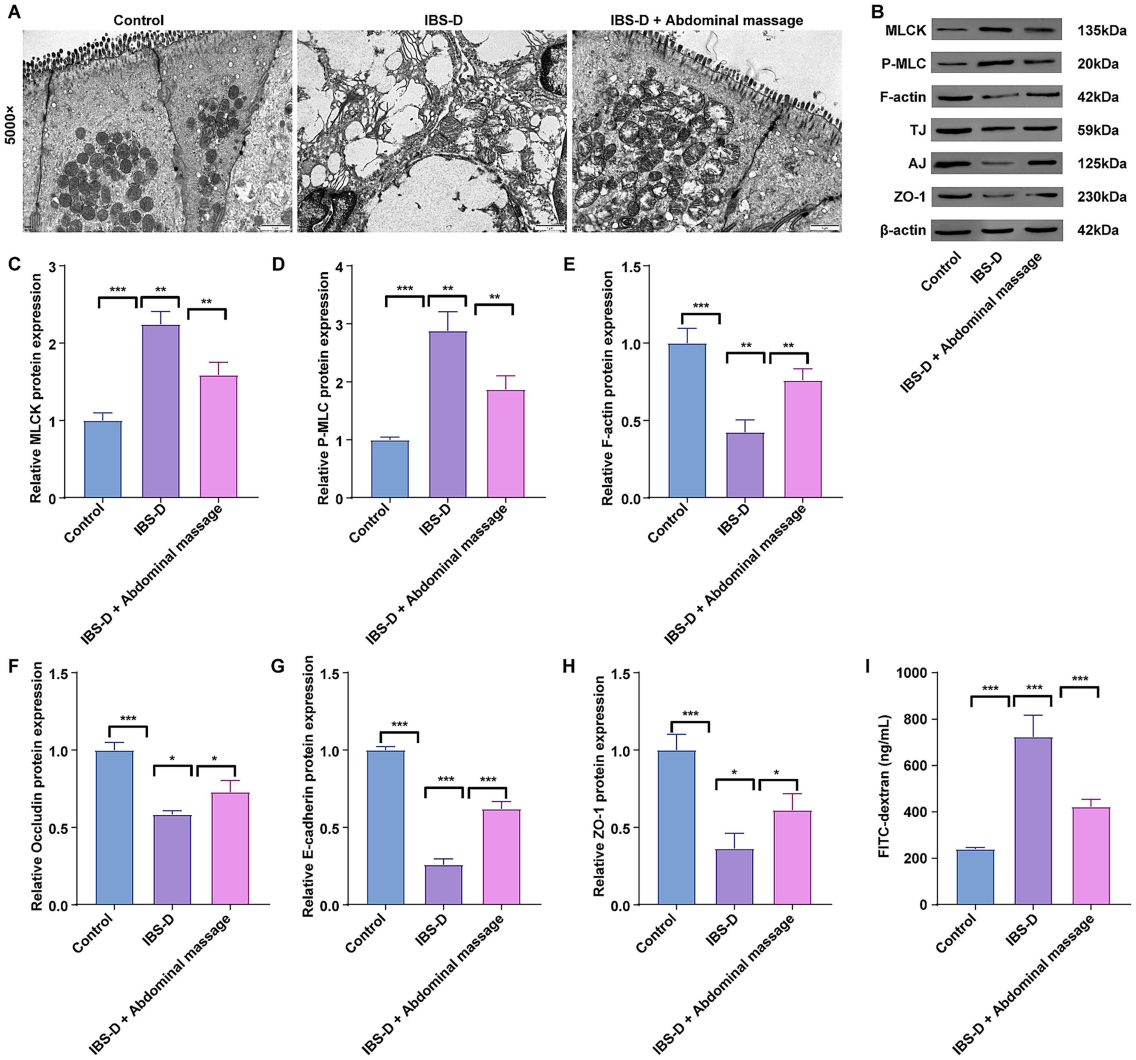
Abdominal massage ameliorates intestinal barrier dysfunction in IBS-D rats. **(A)** Transmission electron microscopy (TEM) analysis of intestinal mucosal tissues in the control, IBS-D, and IBS-D + abdominal massage groups. **(B–H)** The levels of MLCK, p-MLC, F-actin, TJ, AJ, and ZO-1 protein expression in the different groups. **(I)** The serum levels of fluorescein isothiocyanate (FITC)-dextran in the control, IBS-D, and IBS-D + abdominal massage groups. ^*^p < 0.05, ^**^p < 0.01, ^***^p < 0.001.

Intestinal mucosal permeability is a key indicator of intestinal mechanical barrier function and is closely associated with the pathogenesis and progression of IBS-D (Linsalata et al., 2023). Therefore, we next investigated the therapeutic effects of abdominal massage on intestinal mechanical barrier function in a rat model of IBS-D. TEM analysis of intestinal mucosal tissues revealed severe ultrastructural damage in the intestinal epithelial cells of IBS-D rats compared with the control group, characterized by loss of microvilli, disruption of intercellular junctions, mitochondrial reduction, abnormal nuclear morphology, and inflammatory cell infiltration in the lamina propria (Figure 8A). Following abdominal massage intervention, significant restoration of the epithelial ultrastructure was observed, including improved arrangement of microvilli, partial repair of cellular junctions, increased mitochondrial quantity, reduction of cytoplasmic vacuoles, and attenuated inflammatory infiltration in the lamina propria (Figure 8A).

Additionally, compared to the control group, the IBS-D group exhibited significantly elevated protein expression levels of MLCK and p-MLC, along with significantly reduced expression of F-actin, TJ, AJ, and ZO-1 (Figures 8B–H). In contrast, abdominal massage intervention partially restored the expression levels of these proteins in IBS-D rats (Figures 3, 8B–H). Finally, we found that serum levels of FITC-dextran were significantly elevated in IBS-D rats compared to the control group, whereas abdominal massage intervention markedly attenuated this increase (Figure 8I). These results indicate that abdominal massage ameliorates intestinal barrier dysfunction in IBS-D rats by restoring ultrastructural integrity, downregulating key permeability-related proteins, and reducing macromolecular permeability.

## 4 Discussion

IBS-D is a prevalent functional gastrointestinal disorder marked by recurrent abdominal pain, increased stool frequency, and loose/watery stools. Symptoms are commonly triggered by dietary factors, emotional changes, or stress (Huang et al., 2023; Staudacher et al., 2023). Its pathogenesis is linked to brain–gut axis dysregulation, visceral hypersensitivity, altered gut motility, immune activation, and microbiota dysbiosis (Garcia Mansilla et al., 2024), though exact mechanisms remain incompletely understood. Current pharmacological interventions often yield limited efficacy and are frequently accompanied by adverse effects. Traditional Chinese medicine therapies, particularly external treatments such as acupuncture and massage, have demonstrated unique advantages in the management of IBS-D and show potential in alleviating its symptoms (Wei et al., 2022). However, the systematic mechanisms underlying these effects require further in-depth investigation. This study, based on the “gut microbiota–immune–barrier” axis and integrating TCM and Western medicine theories, systematically evaluated its effects in an IBS-D rat model. Multidimensional assessments demonstrated that abdominal massage significantly improved diarrhea, abdominal pain, and anxiety-like behaviors. The mechanisms involved modulation of gut microbiota, inhibition of excessive SCFA production, reduction in LPS release, downregulation of the TLR4/MyD88/NF-κB pathway, and restoration of intestinal barrier function.

According to traditional Chinese medicine (TCM), the etiology of IBS-D primarily involves external pathogen invasion, spleen-stomach deficiency, improper diet, kidney yang deficiency, and emotional disturbances, among other factors (Cui, 2023). The abdomen houses multiple organs, including the spleen, stomach, liver, gallbladder, small intestine, and large intestine, and serves as a critical region for qi movement and food transformation. Stimulating abdominal meridians and acupoints through massage can regulate the flow of qi and blood within the meridians, promoting free circulation and thereby improving visceral function (Xin et al., 2020). This study demonstrated that abdominal massage significantly reduced FMC, Bristol stool scores, AWR scores, EMG integral change rate, and intestinal motility in IBS-D rats, while increasing open field test scores. These findings indicate that abdominal massage can ameliorate visceral hypersensitivity, diarrhea severity, depressive and anxious behaviors, and abnormal intestinal motility in IBS-D rats.

Gut microbiota dysbiosis in IBS-D contributes to visceral hypersensitivity, abnormal intestinal permeability, dysmotility, and impaired mucosal immunity (Barbara et al., 2005; Barbara et al., 2016; Wang et al., 2020). Abdominal massage modulates the intestinal microenvironment and systemic neuro-endocrine-immune network, potentially restoring microbial homeostasis (Sobol, 2022). Consistent with prior findings (Mei et al., 2021), our IBS-D model showed decreased Bacteroidetes and increased Firmicutes abundance, which was reversed by abdominal massage. Additionally, massage increased beneficial *Lactobacillus* and reduced *Lachnospiraceae_NK4A136_group*. Numerous studies have demonstrated that *Lactobacillus* abundance is significantly reduced in IBS patients compared to healthy controls (Liu et al., 2017; Zhuang et al., 2017; Wang et al., 2020). Notably, although abdominal massage modestly reduced Alpha diversity, this change coincided with a significant shift in overall microbial structure toward that of healthy controls (β-diversity) and enrichment of functional genera such as *Anaerostipes* and *Tuzzerella*, suggesting that therapeutic efficacy in physical interventions may be better reflected by microbial community remodeling and functional optimization rather than by Alpha diversity alone. *Lactobacillus* promotes SCFA production through carbohydrate fermentation (Qamar et al., 2024). Altered SCFA levels are linked to impaired barrier function and inflammation in IBS-D (Pozuelo et al., 2015). SCFAs frequently exhibit a state of metabolic imbalance in patients with IBS-D. Notably, findings on SCFA levels in IBS patients have been inconsistent, which may be attributed to differences in sample type, disease severity, and analytical methods. While some studies report reduced SCFA concentrations, multiple studies have documented significant elevations in total SCFAs, propionate, and butyrate in IBS-D patients (Gargari et al., 2018; Tian et al., 2020). The elevated SCFA levels observed in our IBS-D model are consistent with this latter body of evidence. Elevated propionate and butyrate may promote colonic motility via activation of G protein-coupled receptors (GPCRs), thereby contributing to increased bowel movement frequency (Sun et al., 2019). Meanwhile, butyrate has been shown to stimulate MUC2 gene expression and enhance mucus secretion, which helps maintain the physical intestinal barrier (Blaak et al., 2020). Despite the generally acknowledged protective role of SCFAs in intestinal barrier function, IBS-D patients often exhibit impaired barrier integrity. This observation suggests a dual role of SCFAs in IBS-D pathophysiology: on one hand, appropriate levels of SCFAs, particularly butyrate, contribute to barrier maintenance and normal motility; on the other hand, metabolic disturbances in SCFA profiles, such as an altered acetate-to-butyrate ratio, may disrupt their physiological functions and even exacerbate intestinal dysmotility (Kai et al., 2023). Furthermore, the effects of SCFAs on intestinal function are concentration-dependent. For instance, low concentrations of butyrate enhance colonic motility, whereas higher concentrations may inhibit intestinal water-electrolyte absorption and mucin secretion, thereby slowing intestinal transit (Binder and Mehta, 1989; Barcelo et al., 2000). In this study, the IBS-D rat model exhibited significantly elevated fecal levels of acetate, propionate, isobutyrate, butyrate, and total SCFAs. This elevation likely reflects dysbiosis-driven excessive SCFA production, which may also be influenced by the stress-induced model employed in this study. Despite elevated SCFAs, the IBS-D model exhibited impaired barrier function, suggesting that their protective effects may be counteracted by other pathological factors. Moreover, excessive SCFAs may exacerbate diarrhea by activating GPCRs to promote colonic motility. These findings suggest that microbial dysregulation in IBS-D leads to SCFA overproduction, and that imbalances in SCFA composition may compromise their protective effects and exacerbate diarrhea by disrupting intestinal motility, barrier function, and osmotic balance. Thus, SCFAs likely play a context-dependent role in IBS-D, with outcomes determined by their concentrations, proportions, and interactions with the intestinal environment. Further research is needed to clarify the dynamics and mechanisms of individual SCFAs in IBS-D pathogenesis.

Moreover, the FMT group exhibited superior improvements in certain behavioral and motility parameters compared to the abdominal massage group. Several factors may account for this difference. First, FMT delivered a high concentration of modulated microbiota directly via daily gavage, whereas abdominal massage modulates the microbiota indirectly through physical stimulation, exerting a more gradual effect. Second, antibiotic pretreatment in FMT recipients reduced competition from endogenous microbiota, facilitating more robust colonization. Importantly, these findings further support our central hypothesis that the therapeutic effects of abdominal massage are mediated, at least in part, through gut microbiota modulation, as transplantation of massage-modulated microbiota alone was sufficient to replicate, and in some parameters even enhance, the beneficial outcomes. No significant differences were observed between the two groups in most outcome measures (e.g., EMG integral change rate and SCFA levels), suggesting comparable overall efficacy through distinct delivery routes.

FMT from abdominal massage-treated donors reproduced the therapeutic benefits, reducing fecal output, visceral sensitivity, intestinal motility, and anxiety-like behaviors, while also decreasing SCFA levels—particularly acetate and propionate. This confirms that gut microbiota mediates the alleviation of IBS-D symptoms by abdominal massage. IBS-D is also associated with increased LPS biosynthesis and release (Mishima and Ishihara, 2020; Matsumoto et al., 2021), which exacerbates intestinal inflammation and barrier dysfunction (Gao et al., 2022). We found that both abdominal massage and FMT reduced cecal LPS levels. LPS binds TLR4, activating the MyD88/NF-κB pathway and triggering pro-inflammatory cytokine production (Xi et al., 2022). TLR4/NF-κB activation is well-documented in IBS-D patients (Zhang et al., 2024) and contributes to both inflammation and barrier disruption (Han et al., 2017; Roudsari et al., 2019). In the present study, abdominal massage suppressed LPS-induced TLR4/MyD88/NF-κB signaling, thereby attenuating intestinal inflammation in IBS-D rats. Furthermore, NF-κB activation upregulates MLCK, leading to MLC phosphorylation, perijunctional actin–myosin contraction, and internalization of TJ proteins (Yaylaoglu et al., 2006; Yang et al., 2017). This disrupts intestinal barrier integrity, a process amplified by pro-inflammatory cytokines (Mishima and Ishihara, 2020). Abdominal massage inhibited MLCK overexpression and enhanced the expression of F-actin, TJ/AJ proteins, and ZO-1. These results suggest that abdominal massage may improve intestinal barrier function by modulating the TLR4/MyD88/NF-κB/MLCK pathway and promoting structural restoration of epithelial junctions. It is noteworthy that FMT similarly reduced circulating LPS levels and downregulated the TLR4/MyD88/NF-κB pathway along with pro-inflammatory cytokines in recipient animals. These findings, although not based on direct barrier measurements, are consistent with the hypothesis that FMT may improve barrier function through the same LPS/TLR4/NF-κB/MLCK axis, albeit further direct evidence is required to confirm this causal link. While our findings highlight the TLR4/MyD88/NF-κB pathway as a key mediator of the therapeutic effects of abdominal massage in IBS-D, we acknowledge that physical interventions such as massage may also engage mechanotransduction and neuroendocrine pathways, warranting integrated investigations into these mechanisms in future studies.

It is noteworthy that FMT markedly reduced plasma LPS and suppressed TLR4/MyD88/NF-κB signaling and pro-inflammatory cytokines, even though barrier-related endpoints were not directly measured in FMT recipients. These molecules are well-established upstream drivers of MLCK-dependent tight junction disruption and barrier leakage (Cani et al., 2007; Turner, 2009; Chelakkot et al., 2018). Therefore, while our findings are consistent with a microbiota-mediated improvement in barrier integrity via the LPS/TLR4/NF-κB/MLCK axis, they do not directly prove this causal link. Future studies should incorporate direct barrier assessments (e.g., TEM, junctional proteins, permeability assays, and MLCK/p-MLC) in FMT recipients to further validate this mechanism.

Several limitations should be noted. First, the study lacked a procedure-matched sham control for the abdominal massage intervention, which should be implemented using a mechanized device in future work to ensure blinding rigor. Second, ceftriaxone preconditioning was administered only to the fecal microbiota transplantation recipients, introducing a potential confounding variable in intergroup comparisons. Moreover, the independent effects of ceftriaxone on intestinal permeability and inflammatory markers remain unclear, warranting further investigation. Third, the rodent model, while valuable, cannot fully recapitulate the complex psychosomatic interactions characteristic of human IBS-D. Fourth, the observed increase in short-chain fatty acids, which contrasts with certain existing literature, warrants further investigation to clarify their precise role in disease pathophysiology. Fourth, behavioral data suggested a dissociation between pain relief and anxiety improvement, but this study could not determine whether the anxiolytic effect of abdominal massage resulted from physical pain alleviation or direct neural modulation, a distinction requiring future mechanistic studies. Fifth, while the maternal separation combined with chronic stress model effectively recapitulates the brain-gut axis dysfunction and core intestinal pathologies of IBS-D, it does not capture the full etiological complexity of human disease, including genetic susceptibility and dietary factors; findings should therefore be interpreted as mechanistic insights rather than comprehensive etiological simulation. Furthermore, the long-term sustainability of the therapeutic effects and the detailed neuro-immune mechanisms involved remain to be evaluated. Finally, a key constraint is that direct assessments of intestinal barrier structure and function—such as ultrastructure, junctional protein expression, permeability, and MLCK/p-MLC signaling—were not performed in FMT recipients, precluding a definitive mechanistic link between microbiota changes and barrier repair within this experimental module. Future studies addressing these aspects will provide a more comprehensive understanding.

## 5 Conclusion

In summary, abdominal massage reduces inflammation and alleviates IBS-D symptoms by remodeling gut microbiota, reducing LPS release, suppressing SCFA overproduction, inhibiting TLR4/MyD88/NF-κB signaling, and restoring mucosal barrier integrity via MLCK downregulation and enhanced junctional protein expression. This study further elucidates the molecular mechanisms by which abdominal massage alleviates IBS-D via modulation of the microbiota-immune-barrier axis, providing a critical theoretical foundation for non-pharmacological therapeutic interventions.

## Data Availability

The raw data generated in this study can be found in the NCBI (https://www.ncbi.nlm.nih.gov/), accession PRJNA1477205.

## Supplementary material

Supplementary Figure S1 Flowchart of gut microbiota depletion and fecal microbiota transplantation (FMT) Intervention. The figure of the graphical representation was generated by Figdraw (http://www.figdraw.com).

Supplementary Figure S2 Abdominal massage alleviates visceral hypersensitivity, improves gut motility, and reduces anxiety in IBS-D model rats. **(A,B)** The abdominal withdrawal reflex (AWR) scores and electromyographic (EMG) integral among the control, IBS-D, and IBS-D + abdominal massage groups under different colorectal distention (CRD) pressure levels at post-modeling. **(C)** The fecal number, rectal bead expulsion time, and fecal weight among the control, IBS-D, and IBS-D + abdominal massage groups at post-modeling. **(D)** The number of instances of standing, grid crossings, entries to the central area, and the time in the center among the control, IBS-D, and IBS-D + abdominal massage groups at post-modeling. ****p* < 0.001.
