# Supplementary figures and images for: Abdominal massage alleviates IBS-D by modulating the gut microbiota and suppressing the LPS/TLR4/NF-κB/MLCK pathway
Source: Front Microbiol. 2026 Jun 26;17:1730607. doi: 10.3389/fmicb.2026.1730607 (PMC13353101; doi:10.3389/fmicb.2026.1730607)

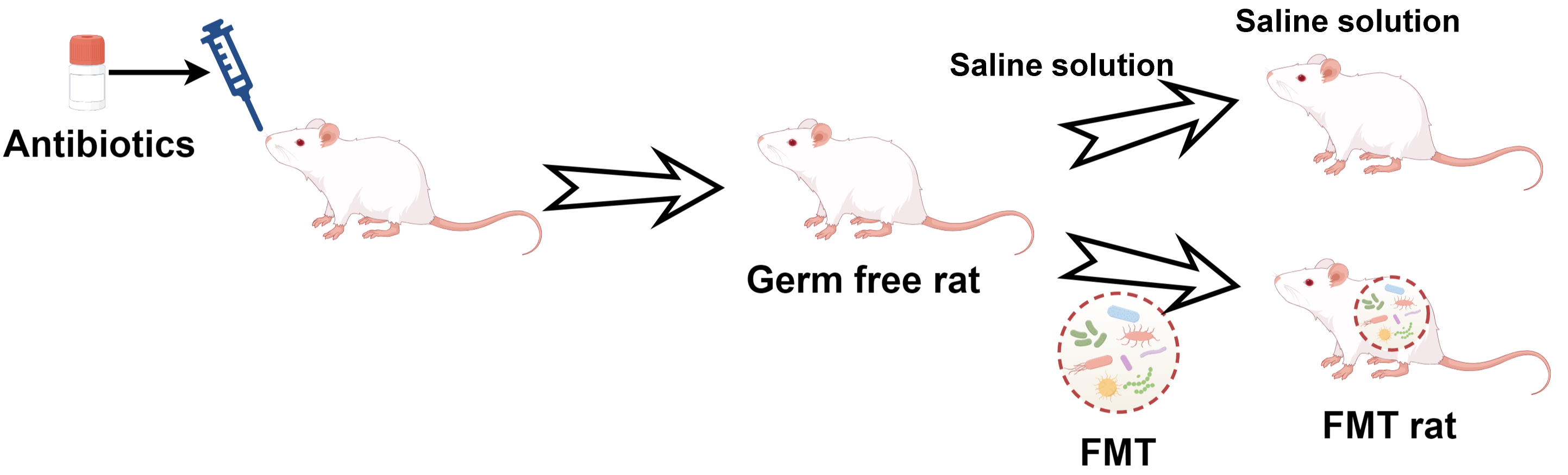

Supplement: Supplementary Figure S1 — Flowchart of gut microbiota depletion and fecal microbiota transplantation (FMT) Intervention. The figure of the graphical representation was generated by Figdraw (http://www.figdraw.com). [file Image_1.TIF]

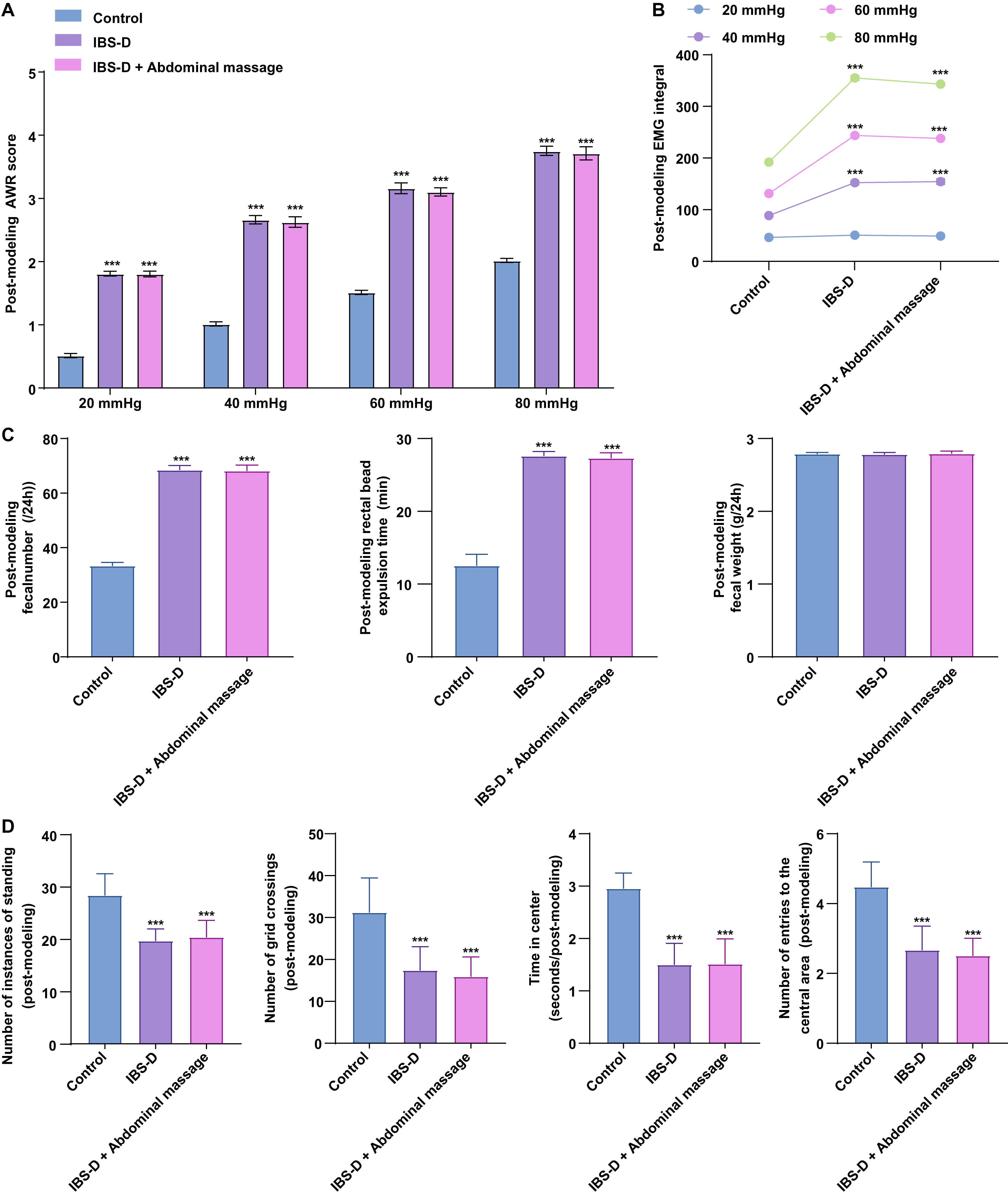

Supplement: Supplementary Figure S2 — Abdominal massage alleviates visceral hypersensitivity, improves gut motility, and reduces anxiety in IBS-D model rats. (A,B) The abdominal withdrawal reflex (AWR) scores and electromyographic (EMG) integral among the control, IBS-D, and IBS-D + abdominal massage groups under different colorectal distention (CRD) pressure levels at post-modeling. (C) The fecal number, rectal bead expulsion time, and fecal weight among the control, IBS-D, and IBS-D + abdominal massage groups at post-modeling. (D) The number of instances of standing, grid crossings, entries to the central area, and the time in the center among the control, IBS-D, and IBS-D + abdominal massage groups at post-modeling. ***p < 0.001. [file Image_2.TIF]
