# Supplementary material for: Abdominal massage alleviates IBS-D by modulating the gut microbiota and suppressing the LPS/TLR4/NF-κB/MLCK pathway
Source: Front Microbiol. 2026 Jun 26;17:1730607. doi: 10.3389/fmicb.2026.1730607 (PMC13353101; doi:10.3389/fmicb.2026.1730607)
Supplement: Supplementary file 3 [file Image_3.pdf]

The original WB of Figure 7B

MyD88

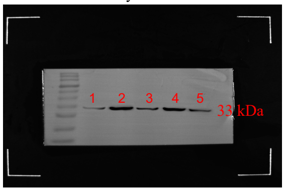

NF-κB

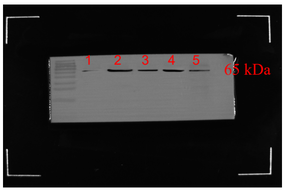

TLR4

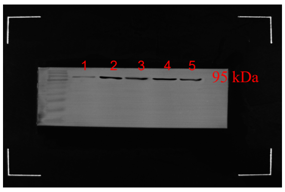

β-actin

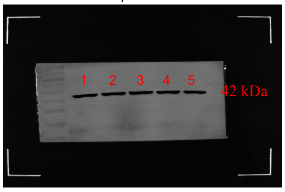

1: Control
2: IBS-D
3: IBS-D + Abdominal massage
4: IBS-D + Sham massage
5: IBS-D + FMT

The original WB of Figure 8B

AJ

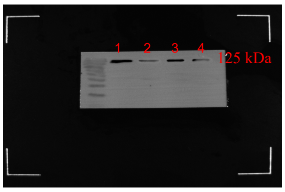

F-actin

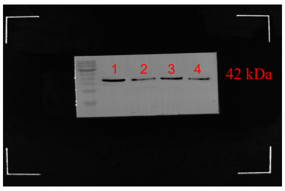

MLCK

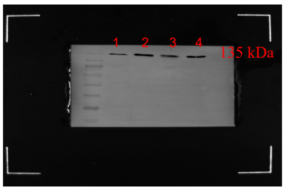

p-MLC

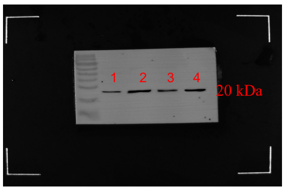

ZO-1

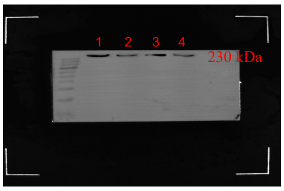

TJ

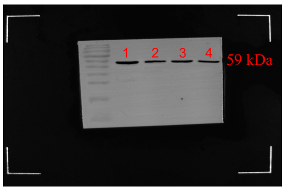

β-actin

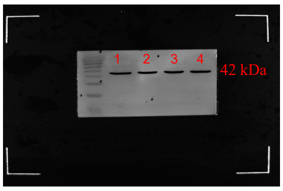

1: Control
2: IBS-D
3: IBS-D + Abdominal massage
4: IBS-D + Sham massage

The experimental data for the IBS-D + sham massage group, although obtained during the Western blot analysis, was not presented in the results section or figures of this manuscript.
